# Learning from “Knocks in Life”: Food Insecurity among Low-Income Lone Senior Women

**DOI:** 10.1155/2012/450630

**Published:** 2012-09-11

**Authors:** Rebecca J. Green-LaPierre, Patricia L. Williams, N. Theresa Glanville, Deborah Norris, Heather C. Hunter, Cynthia G. Watt

**Affiliations:** ^1^Department of Applied Human Nutrition, Mount Saint Vincent University, Halifax, NS, Canada B3M 2J6; ^2^Atlantic Health Promotion Research Centre, Dalhousie University, Halifax, NS, Canada B3H 3J5; ^3^Department of Family Studies and Gerontology, Mount Saint Vincent University, Halifax, NS, Canada B3M 2J6; ^4^Participatory Food Costing Project, Mount Saint Vincent University, Halifax, NS, Canada B3M 2J6

## Abstract

Building on earlier quantitative work where we showed that lone senior households reliant on public pensions in Nova Scotia (NS), Canada lacked the necessary funds for a basic nutritious diet, here we present findings from a qualitative study involving in-depth interviews with eight low-income lone senior women living in an urban area of NS. Using a phenomenological inquiry approach, in-depth interviews were used to explore lone senior women's experiences accessing food with limited financial resources. Drawing upon Bronfenbrenner's Ecological Systems Theory, we explored their perceived ability to access a nutritionally adequate and personally acceptable diet, and the barriers and enablers to do so; as well in light of our previous quantitative research, we explored their perceptions related to adequacy of income, essential expenses, and their strategies to manage personal finances. Seven key themes emerged: world view, income adequacy, transportation, health/health problems, community program use, availability of family and friends, and personal food management strategies. World view exerted the largest influence on seniors' personal perception of food security status. The implications of the findings and policy recommendations to reduce the nutritional health inequities among this vulnerable subset of the senior population are considered.

## 1. Introduction

In 2008, we published unique work addressing the question of whether Canada's public pensions, Old Age Security ^1^ and the Canada Pension Plan ^2^, provide adequate income for four hypothetical senior households living in Nova Scotia (NS) to afford a basic nutritious diet [1]. This was the first published study using food costing ^3^ data to investigate the sufficiency of public financial programs targeted at seniors. Results showed that single-member households in NS in 2008 lacked the necessary funds, with monthly deficits estimated to be as high as $224.18 if they were to purchase a basic nutritious diet. 

The province of NS is home to the oldest population in Canada, with 16.5% of its residents over the age of 65 years; the majority of which are women [2]. As elsewhere [3], senior women in NS are disproportionally impacted by poverty. In 2006, almost one-third of lone senior women lived below the before-tax low-income Cutoff (LICO)^4^ Canada's unofficial poverty line, compared to one in five men who lived alone [4]. In NS, 74% of seniors living below the LICO are women [4]. 

## 2. Purpose 

The purpose of this paper is to explore the phenomenon of food insecurity for low-income lone senior women living in NS. This study used in-depth interviews to explore lone senior women's experiences accessing food with limited financial resources. We explored their perceived ability to access a nutritionally adequate and personally acceptable diet, and the barriers and enablers to do so; as well in light of our previous quantitative research [1], we explored their perceptions related to adequacy of income, essential expenses, and their strategies to manage personal finances.

## 3. Background

The United Nations' Food and Agricultural Organization states that “food security exists when all people, at all times, have physical and economical access to sufficient, safe, and nutritious food that meets their dietary needs and food preferences for an active and healthy life” [6, page 1560]. An assured ability to acquire acceptable foods in socially acceptable ways is also considered a key component of being food secure [7]. The easiest way to understand food insecurity is lack of financial resources to purchase food needed and wanted; however, in some of the pioneering research, which came out of Cornell University, on hunger and food insecurity, Radimer and colleagues [8] found that two dimensions of food insecurity existed: an individual dimension and a household dimension with each dimension containing four components: quantitative, qualitative, psychological, and social. 

The quantitative and qualitative components of food insecurity are the most easily measured: experiencing hunger, going without food, or having to eat less food than usual, and consumption of nutritionally inadequate meals. Individuals reporting food insecurity are significantly more likely to consume less energy and other key nutrients than those self-reporting as having sufficient food [9, 10]. The psychological component to food insecurity is exemplified by an individual's anxiety over their food situation, such as wondering where their next meal will come, while the social component is manifested by socially or culturally less normative patterns of eating (e.g., skipping meals), and acquiring food in socially unacceptable ways (e.g., using a food bank) [8]. While these characteristics have been documented among senior populations in the US by Wolfe et al. [5], they also found that having the right foods for health and anxiety over not having these specific health-related foods are phenomena within the qualitative and psychological components of food insecurity that are unique to seniors. 

Other food security research focusing on seniors has also found that finances are not the only limiting factor for seniors to access acceptable and adequate food. Upon analyzing in-depth interviews with 145 adults, age 70 years and older, living in rural North Carolina, Quandt and Rao [11] revealed that barriers to food security in rural American seniors were associated with material (e.g., low-income), social (e.g., limited family network), and health (e.g., presence of disease or disability) related factors. Dean et al. [12] report that among older and senior adults in a largely rural area of central Texas, food insecurity is associated with diminished social capital (limited access to community and familial resources) and perceptions of personal disparity in comparison with others. Single, widowed, or divorced persons also reported higher levels of food insecurity, related in part to limited familial social capital [12]. Reliance on others for help with food-related activities such as grocery shopping and meal preparation can influence food intake and can be considered part of the concept of food insecurity among older adults [13]. 

There is also the “generational lens” through which seniors view the world. Other work completed by Quandt and colleagues [14] suggests that pride, self-sufficiency, and “you cannot always get what you want” attitude are aspects of the generational lens that colour the nature of food security for seniors. These “senior-relevant” factors highlighted by researchers in the US suggest that what food security means to seniors, and therefore how they manage their ability to access food is different than younger populations' views of food security. However, Canadian research in this area is lacking. Given the different history and health and social systems in the US, it is not clear whether pride and a self-sufficient attitude would play the same role in a Canadian senior's meaning of food security. 

These newer “senior-relevant” factors also suggest the possibility that Canada's current food security measurement tools, which focus on low-income as the most significant determinant of food insecurity [15], may not accurately capture the other major enablers and barriers seniors face in accessing sufficient, quality foods in socially acceptable ways. It is possible we are not understanding and therefore not capturing the full picture of food insecurity among seniors. 

The negative relationship between food insecurity and one's ability to access a nutritious diet and mitigate risk for chronic disease is well understood. Chronic diseases commonly impacting seniors such as cardiovascular disease and cancer are used to exemplify the relationship between insufficient access to nutritious food and the resulting poor health outcomes [16–18]. Therefore, understanding how a particular group of seniors—lower income senior women living alone in Nova Scotia—attempt to maintain food security, can provide insights to the necessary policies and programs needed to reduce the nutritional health inequities among this vulnerable subset of the senior population.

## 4. Theoretical Framework

An ecological model was used to frame the participants' perceived realities regarding their ability to access a nutritionally adequate and personally acceptable diet, the barriers and enablers to do so; as well their perceptions related to adequacy of income, essential expenses, and their strategies to manage personal finances, guiding our approach to addressing our research purpose. Ecological models reveal that individual behaviours such as food procurement patterns are influenced by biological, demographic, psychological, social/cultural, environmental, and policy variables. Bronfenbrenner's Ecological Systems Theory [19, 20] was used as a framework to examine the many different areas in the seniors' environment where these variables exert their influence. 

Three key themes characterize ecological systems theory. First, individuals are characterized as embodied within a system comprising a nested arrangement of structures. The first of these structures, the microsystem refers to the face-to-face interactions within immediate settings such as home, neighbourhood, and informal social networks. The mesosystem comprises the relationships between and among the immediate settings, for example, family, seniors' centres, and community facilities. The exosystem consists of the social structures, both formal and informal, removed from the individual but yet functioning at the local level to regulate and control their everyday lives within the *microsystem. *These structures include the major institutions within society rendered visible through the implementation of policies and practices at the local level. Examples include mass media, government agencies, and social networks. *Macrosystems *may be defined as the overarching meaning systems, conveyed symbolically through culture. Macrosystems such as religious, political, legal, economic, health, and social systems are understood in ecological theory not just as reified structures but also as carriers of ideology that construct a “world view” that, both implicitly and explicitly, shapes experience at all other levels of the environment [21, 22]. 

Second, the conceptualization of the levels of the environment as a *nested *arrangement of structures implies that they are not mutually exclusive. Rather, it is assumed that a system cannot be understood when broken into its component parts or when separated from its context. Transactions flow bidirectionally from the outer level (*macrosystem*) inward, and conversely, from the inner level (*microsystem*) outward. These reciprocal exchanges are vital to maintaining the equilibrium of individuals, a proposition that has significance for this research. 

Third, ecological theory brings into view the *interdependencies *that exist between immediate environments and broader systems. Change in one part of the system will stimulate changes in other parts. An ecological approach facilitates and emphasizes consideration of the impact of multiple and overlapping influences in addressing food security issues. 

## 5. Methods

### 5.1. Data Collection and Sampling

Eligible participants were women 65 years of age or older, who lived by themselves in a noninstitutionalized setting (i.e., independent) in urban NS, and who were in receipt of the Guaranteed Income Supplement (GIS)^5^. Women living by themselves who had a spouse in a nursing home, hospital, or other type of institution were ineligible to participate. Interviews took place over the course of four months between June and September 2007. Lone senior women were the focus of this research as national statistics show this subgroup of the senior population at greatest risk for poverty and the most likely to not access all the financial benefits available [4, 23], and our previous research suggest seniors living alone are more likely to be unable to afford a nutritious diet than those living with a partner [1]. Sampling was done using purposive and snowball methods, using site-based recruitment through health care agencies and seniors' centres. Data collection and analysis continued concurrently until theoretical saturation was reached at eight participants.

Women participated in semistructured face-to-face interviews. The interview guide used to facilitate the interview was formulated using Radimer's conceptualization of food insecurity and hunger with its individual and household dimensions and four components: quantitative, qualitative, psychological, and social [8]. The intent was to use this framework to draw out health and world view as distinguishing features of food security in the elderly. The interview guide also contained questions developed by the Institute for Research on Poverty [24] dealing with how study participants procured and prepared food, their typical daily food routine, and asked questions about a time (if relevant) when they had difficulty getting enough food. Lastly, the interview guide asked participants to review hypothetical affordability scenarios comparing monthly income to expenses constructed in our earlier work [1] to gain their perspective on adequacy of Canadian public pensions to afford the food they needed and wanted, as well as other essential expenses. Broad, open-ended, semi-structured questions with probes were used.

Interviews took place in participants' homes or a location of the participant's choosing. Interviews were conducted in English, lasted between 60 and 120 minutes, were digitally recorded and transcribed verbatim. Journal and field notes were also recorded directly following the interviews. All participants were offered the opportunity to review their transcript. Study procedures were approved by Mount Saint Vincent University's Ethics Review Board. 

### 5.2. Data Analysis

During the analysis, a phenomenological approach [25], in combination with a conceptual framework based on Bronfenbrenner's ecological model [20], was used to explore senior women's perceptions of their lived realities. Phenomenology is used to uncover the meaning behind a phenomenon, it goes beyond simply describing an experience, seeking to arrive at a structural description of the experience and expose the underlying and precipitating factors that account for what is being experienced [26]. 

Transcribed interviews, journal and field notes were imported into NVivo 7.0, a qualitative data analysis software program (QSR International, 2007). One of the authors (RGL) analyzed transcripts by systematically coding text, informed by the interview guide topics and themes that emerged from the data corresponding to environmental levels in Brofenbrenner's Ecological Systems Theory (microsystem, mesosystem, exosystem, and macrosystem) [19, 20]. Later, findings and links to the conceptual framework were strengthened through investigator triangulation.

## 6. Results

### 6.1. The Participants

Of the eight women who participated, four were 65–74 years of age and four were 75 years or older. All women rented their dwelling: one lived in a duplex, one in a townhouse complex, one in an apartment building, and five lived in senior-geared apartments. Seven of the eight women lived in subsidized housing, meaning their rent was based on a percentage of their income, while the eighth participant paid market rent for her duplex. One woman was never married while the other participants lived alone because they were either widowed or divorced. All women received the basic OAS pension and GIS. Four women received a Canada Pension, the result of their contribution to the Pension Plan when they were previously employed. Two women received survivor's benefit through the CPP. Only one woman reported receiving a private pension, although it only amounted to $73/month after working almost 25 years. None of the women owned a car; health reasons and finances were the main impetuses for “giving up” their vehicle. 

**Figure 1:**
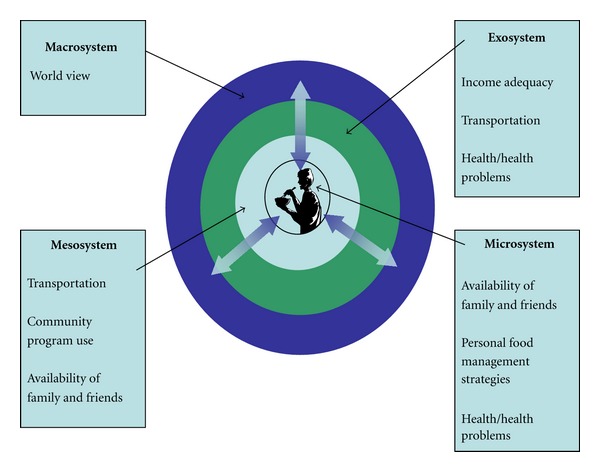
How low-income lone senior women living in NS experience food security, organized according to Bronfenbrenner's Ecological Framework.

As the women talked about their experiences managing finances, accessing the food they needed and wanted, and how they viewed their situation, seven main themes emerged depicting their perception of their food security status. These themes can be placed within the four interconnected layers of the seniors' environment as defined by Bronfenbrenner: (1) at the macrosystem level, *world view, *(2) within the exosystem, *income adequacy*, (3) at both the exo- and mesosystems levels, *transportation related factors,* (4) at both the exo- and microsystem levels, *health/health problems, *(5) within the mesosystem*, community program use*, (6) at the meso- and microsystems, *availability of family and friends,* and (7) within the microsystem,* personal food management strategies*.  Figure 1 provides a visual map of the meaning of food security to these women, organized according to Bronfenbrenner's Ecological Framework. 

### 6.2. World View (Macrosystem Factor)

All of the women in this study met the criteria (according to its definition) to be classified as food insecure. They all used various coping skills to bring food into the house, such as accessing food banks, stretching meals, relying on credit; however, interestingly, none of them perceived themselves to be food insecure. At one or more points in the interview, participants were asked if they could recall a time in their life since they were a senior and living on their own when they had difficulty getting the food they needed and wanted. Some shared stories of going hungry in the past, such as when they were caring for their children or when they were first on their own without their spouse, but none considered their current situation as food insecure. A world view, or generational lens, develops as a result of past life experiences, cultural and religious beliefs and personal belief systems, and contribute, to the women's self-perceived food security status.

These women had a general contentedness about their diet, their life, and general circumstances. As stated by one participant: “*No, because as I say I've been doing good the way I've been doing now for many years and I've got no complaints with any of it. I've got a roof over my head and I've got food to put in my stomach and my doctors tell me I'm doing good so there's nothing else I could want. I mean there's a lot of people that can't say that.*” Although this woman was content with her life, she accessed a nearby food bank frequently, sometimes weekly. Despite needing to access food in a socially unacceptable way on a regular basis she perceived nothing problematic with her situation—due to her resourcefulness she did not lack food. A sense of resilient self-sufficiency was detected in the women interviewed, as most spoke about needing to be vigilant about their spending to assure that bills could be paid and assuring they had enough for the expenses they needed versus what they wanted. It was evident in talking with them that they had been through a lot and could manage most things that might come their way. “*I had a hard time bringing up my children, and you learn from your knocks in life eh? Don't want to go through those again, I got to look after me, and I am, I'm looking after me.*”

The lives all of these women were impacted in some way by the Depression and World War II so they understood the need to be resourceful and not wasteful. Some were lone parents, as were these two participants: “*No I've never ever went hungry, the only time I've ever went hungry when I was with my children*”. “*Because I went for quite a few years where I didn‘t get enough to eat because I had to make sure my children were eating.*” Experiencing hunger earlier in life shaped how they saw their current situations; by definition, the participants experienced food insecurity within the quantitative component.

All eight women acknowledged factors hindering their access to food; however, they perceived the strategies they took to overcome the obstacles as either acceptable or just a fact of life; just something you do if you're old, poor, and so forth. None of the participants identified themselves as living in poverty or being in great need, or even want. The generational lens through which they viewed their situation allowed them to compare their current situation to situations in the past where they faced extreme financial difficulties; relative to more difficult times, they perceived their current income to be adequate, in other words, their perspectives were colored by their prior experiences and resultant world view.

### 6.3. Adequacy of Income (Exosystem Factor)

As the women reviewed the affordability scenarios created in our previous work that compared monthly income to monthly basic expenses for two hypothetical lone senior households [1], the women were very candid in disclosing how much income they received each month and what bills their income went towards. The biggest lesson from their stories was the role of GIS and income-geared housing in protecting the funds remaining for food, and the importance of a “med-alert” type device and cable TV, two expenses which the participants deemed to be essential. 

In general, the women were appreciative of the pensions they received, they were thankful to have a reliable monthly income; however, there were notions that they could use a bit more. “*Old Age Pensions and that? Well I tell ya, they could be more. They could be more. Of course, now this is a God send, what I get now compared to what I got then…when I was on mothers' allowance.*” Another participant shared: “*Oh, I'm always just about at the end of it at the end of the month … nobody's gonna get rich when I go, there'll be no fighting over my money!*” Public Pensions programs exist at the exosystem level, they are formal programs removed from the individual, but yet functioning at the local level to regulate and control their everyday lives and ability to access food.

Another significant example of a social structure directly impacting seven of the eight interviewed women's lives was the provincial senior social housing program. Two of the participants explain: “*Every year you have to call Ottawa and get them to send you a letter saying exactly what you're getting then you give that to Housing and they figure out 30% of it, they take. And the rest you've got for your TV and your phone and your groceries and to live on. It doesn't leave you a lot … Before you get your Old Age pension you only pay 25% of what you're getting, but once you hit 65 you pay 30% …*”

“*[Housing] always take a third because that's their share of the rent. Every time rent comes up they take a third of that, whatever you get, so they can't put any more on for rent, because it's subsidized housing.*” This controlled rent expense meant despite the adequacy or inadequacy of their overall income, seven of the eight women never had to pay a disproportionate amount on shelter; thus their funds could be better distributed to other necessary expenses. 

While none of the women conveyed that their food intake suffered because of inadequate income, due to their various coping strategies, what did seem to suffer was their social life. For example, participants commented, “*Like I never go to movies, the last time I went to a movie I took my children by their hands to see Herbie The Love Bug. They're now 46 and 43. Because I can't afford to go to movies.*” or “*No I can't afford to go out [to dinner], unless one of my children, my daughter she generally takes me out on Mother's Day and on Christmas I generally go down to her place … but no I eat by myself.*”

Although admittedly it wasn't a necessity, cable TV was viewed as an important component of their daily routine and helped to address the isolation these women experienced. Many watched television while eating their meals alone, and many would watch their favourite shows to help pass the time. “*If you don't have cable you can't watch TV … like I've got digital cable because I can't go out. I mean the girls will come in to play cards and sometimes we'll go to Bingo every once and a while but that [cable] is the only enjoyment I have, know what I mean?*” Within the context of these low-income lone seniors' lives, cable was essential to help them cope with their situation, to help improve their quality of life.

### 6.4. Transportation (Exo- and Mesosystem Factor)

The cost and feasibility of transportation was a major reoccurring theme during all interviews. None of the women owned cars, many had mobility issues, and all depended on family, friends, or neighbours to access grocery stores, medical appointments, or church. 

Even the municipal accessible bus service did not meet the needs of some of the participants to access the foods they needed and wanted. Changes in the bus routes and a policy stating the accessible bus would only venture a certain number of meters off the urban transit (nonaccessible) route meant one participant could no longer rely on this service. Another participant noted how far in advance she would have to schedule the bus. “*You have to give exactly two weeks notice and then they'll work you in. So therefore you have to make your appointments [well in advance] … And you [referring to interviewer] can get up in the morning and say I think I'll go get groceries today … we can't do that.*” 

Within the mesosystem layer of the women's environment, family and friends provided the transportation to enable access to food for six of the eight women. The senior woman is involved in her mesosystem environment albeit not as intensely, as she will not have as much control at this level because it will always involve influence from another party (e.g., the family member or friend). While family and friends were the primary source of transportation for six of the women, for others family members were dispersed across the country, or of those that lived nearby some were unable or unwilling to provide this service for the participants on a consistent, reliable basis. 

### 6.5. Health and Health Problems (Exo- and Microsystem Factor)

A myriad of health problems affected this group of women: five suffered from arthritis or joint pain, four had Type 2 diabetes, four had hypertension, three had some type of digestive disorder, three had full or partial dentures, two were on medication for dyslipidemia, and two were cancer survivors. Stroke, thyroid problems, cardiovascular disease, and severe food allergies were reported once each. Overall, the main health problems contributing to food insecurity were physical and mobility limitations. The participants' multiple health issues required various medications, thus resulting in these women being regulated by drug plan policies. For six of the women enrolled in the provincial seniors' Pharmacare ^6^ program, they greatly benefited from the policy that exempted all seniors in receipt of the GIS from paying the annual premium associated with Pharmacare. The copayment policy also protected them from paying extreme costs at the pharmacy—the plan is structured so seniors pay a certain percentage of the total prescription cost at the pharmacy up to a maximum amount of (at the time of study) $30 for each prescription, with an annual maximum of $382. One participant explains: “*We gotta pay 30% … it doesn't matter how much my medication comes to, you still only pay $30. Now this here [referring to a prescription], it's $96.95 but you still only pay $30. And same with my cholesterol pills, you still only pay $30. Those cholesterol pills are $133.82. But I still only pay $30. It doesn't matter if they were $500, you'd still only pay $30.*”

Saving money on medications and health benefits was important so that money could be better distributed to other parts of the budget, despite loss of services. One of the participants, formerly with her deceased husband's employer's insurance company, shared that once she turned 65 years of age and was eligible for Pharmacare she took advantage of the cheaper drug insurance plan, despite losing some health services. “*Yeah, I dropped Blue Cross; the thing was costing me a lot of money. But I used to be able to get some therapy for my arthritis, they'd pay for that but Pharmacare don't pay for that.*”

Throughout the interviews, as the women reviewed the affordability scenarios created in our previous work [1], an emergency response-type service was often mentioned as an essential expense, where the client wears a button, as a pendant or bracelet, which activates a speakerphone putting them in touch with the operator who in turn phones a first responder. A couple of the participants shared stories of how this service has alleviated the fear of having a fall when living alone. “*I call it my panic button. Oh I've used it a lot.*” One woman was adamant that it should be built into the Pharmacare program. “*I think not enough have it. I think that should be a basic thing that you should have to have. Some before they turn 65 if they have any type of medical problem and it should be required when you turn 65.*”

Grocery stores were another social structure which the women had minimal control over as store location and layout greatly impacted their food access. While grocery shopping had previously been viewed as a social outing for some of the women, mobility limitations turned it into a chore. “*Oh it's too much walking around. The stores are too big and my foot's too sore, I could never get around it. You know, just like going to the mall, you know, the mall is so awful, you know about that, the mall, it's so big by the time you get around and get home it's just like, ahhhh. You feel like you're dead. And grocery stores are just the same, they're too big and I don't want to get in no wheelchair, where am I gonna put the groceries at?*”

Health and health problems were also a direct part of the women's microsystem, a part of their everyday experiences interacting with their diet. For example, needing to avoid food because of allergies, sensitivities or because it was contraindicated with a medication was an issue for some of the women. One participant had nine different ingredients listed on her allergy information card, meaning sometimes she was left with little choice for meal options available through the congregate dining program at her seniors' manor. “*Your only choice is take it or leave it. A lot of foods I'm allergic to and they used to substitute but now the new the director's decided no no, no more substitution …. I'm allergic to many, many foods. I'm allergic to hamburg, which (participants of the congregate dining program) eat a lot. And things like peas, and carrots and bananas, and all kinds of nuts …*” The reality for this particular woman was that her health issues dictated whether she could participate in a meal or not, thus comprising her nutritional intake, her social interactions from being part of a congregate dining program, and her finances as she lived at a manor where she opted for her monthly rent to include the dining program.

### 6.6. Community Program Use (Mesosystem Factor)

All eight women described how they regularly accessed community-based food resources, including food banks, congregate seniors' dining programs, and meal delivery programs. While home-delivered meal programs have potential to make a significant contribution to the nutrient intake of seniors, it was common for the women in this study to stretch one meal to make it into two, as this participant shared: “*Well at first when I first was getting [a home meal delivery service] they were wonderful. And I always had the soup for my lunch and I could make the dinner do two dinners.*”

Three of our participants regularly accessed a food bank, these women talked casually about their use of food banks with no sense of shame detected, perhaps attributable to life experiences and “world view.” They spoke accolades about the staff and volunteers at the food banks they attended and were so appreciative of the food they received. Not only could they receive food when in need, their food banks also delivered to them, which was considered a “God-send” for one participant who had difficulty walking. “*Well sometimes like if I need, say if I need onions or anything like that, I'll phone up and see if they've got any and if they have well [volunteer] will bring it down*”. They were careful to point out that they only received food when it really needed it and took strides to make their food last as long as possible. “*I just get the food once a month, they give me quite a bit, it lasts, I'm not that big of an eater … they give me stuff that I make on my own, and I make enough that it lasts me for almost the week …*” Throughout the interviews it was clear these women used community food resources when needed and were thankful for the increased access to food they provided.

### 6.7. Availability of Family and Friends (Meso- and Microsystem Factor)

All participants were asked what the biggest factor in their lives was influencing their ability to access food; four of the women immediately responded with “family”. The role that family members played varied amongst the participants, but critical roles were seen in providing transportation to shopping and medical appointments, housework, caring for them if they were sick, tending to legal matters, paying certain bills if necessary, and offering love and company. 

One of the women when asked what was the biggest factor influencing her ability to access food she quickly responded “neighbours with cars,” she explains: “*Well actually I'm in a very lucky situation, because a downstairs neighbour takes another neighbour for groceries every month and she said to me one day I might as well get your groceries while I'm walking around with her. And I just give her my debit card and my list and she does my shopping … And she not only goes and gets my groceries, but she brings them back and she puts them away for me.*” It was unclear if this strong trust between these two individuals existed prior to the woman being unable to shop for her own groceries, or if the trusting relationship was created out of necessity so the participant could bring food into her house.

Three of the women had no family close by. One brought up the point several times throughout the interview that when you have no family, no one to fall back on, you have to be financially prepared for the unexpected. “*It's good to have a little bit of money, it doesn't matter how young you are [and] it don't matter how old you are, you don't know what's going to come up that you're not counting on, and then when you've got nobody to come back on. [I've made wise choices] with the money, it's because I've got nobody to come back on. You've got to have some money or somebody to help you out you know.*”

Social capital, the number of people who can be expected to provide support and the resources those people have at their disposal [27], seemed essential for all women to help them complete various activities of daily living. As age brought about increases in health and mobility issues, reliance on others also increased.

### 6.8. Personal Food Management/Coping Strategies (Microsystem Factor)

Additional to using organized community-based programs, the women in this study employed other food management strategies at the individual level to buffer food insecurity. Strategies included tight budgeting, “stretching” food, stock-piling nonperishables, eating poor quality (old) produce, purchasing food or medications on credit, and using various resources available at grocery stores. A unique shopping practice carried out by one of the women included asking produce department staff to chop or peel vegetables, particularly hard root vegetables like squash and turnip. She would also request staff to cut whole vegetables in half. For example, she knew she couldn't eat a whole bunch of celery and didn't want to pay for something that was going to rot in her fridge. “*I'll say (to produce department staff) I really don't want to buy that great big bunch of celery, because it'll rot on me. And he'll say, So? And I'll say, cut it in half! And if I buy a squash I'll say will you cut my squash, and he'll say how do you want it? And I'll say in quarters. And they'll cut it for me. And if I buy a turnip because my hands can't do it anymore and I'll say will you cut that? How do you want it? Cut it in chunks so I can just peel it and that's what I do … I thought, what's the sense of me buying it if I can't cut it? … Yeah, when you're a senior and you're living alone you got to, economically you got to do it. I got sick of throwing vegetables out. Well I'm not doing it anymore, because I don't have the money.*” The women interviewed were all very resourceful, and perhaps due to their past experiences, current financial restraints and “world view”, they have developed unique strategies to cope with hunger.

## 7. Discussion

The purpose of this work was to explore the phenomenon of food insecurity for low-income lone senior women living in NS. To help us examine the meanings behind these women's experiences of accessing food, Bronfenbrenner's Ecological Systems Theory provided a model to observe the women in their immediate environments and then examine their situations more broadly so as to determine how their everyday actions (e.g., food acquisition and consumption) are mediated by more distal aspects of their physical and social milieus.

Of particular interest was the generational lens that was cast over all the participants' perceptions of their food security status. Despite participants clearly meeting criteria for food insecurity, for example, some reported having difficulty accessing food because they are in too much pain to shop, needing to use food banks or other charitable food models, or having difficulty accessing food that meets special dietary requirements. They also reported needing to make sacrifices such as switching insurance plans or sacrificing social outings so that they can maintain enough self-sufficiency to consider themselves food secure. Yet overwhelmingly, the participants did not self-identify as having any real problem accessing food. This finding may be a product of their world view, a macrosystem factor, which reflects their experiences of living through more difficult, or food insecure periods of life. 

In contrast to findings by Hamelin et al. [28] and our previous work with low-income lone mothers [29–31], the shame often associated with food insecurity and accessing food banks does not seem to be part of the experience for these senior women. American researchers have suggested that experiences are colored by the world view or generational lens which enables seniors to endure hardships, this may be due to the impact of remembering the Great Depression or the World Wars [32, 33]. Similar to findings among seniors in the US, our research suggests that while food insecurity among lone senior women in Canada does exist, senior women's self-perceptions of food insecurity are influenced by a macrosystem cultural lens from an older generation, which may be at odds with current cultural understandings of food insecurity. The finding that senior women in this study have skills and perceptions that enable them to cope with food insecurity raises questions about how future generations will experience food insecurity and how this will impact their health and well-being if they do not share a similar world view or generational lens then that of their parents or grandparents. While an argument can be made that senior women of the future will not be as vulnerable to food insecurity because many more will have worked (may have greater access to CPP or personal savings) and can drive (may not be as isolated), there is a risk that food insecurity among seniors will become a more prominent social issue if we do not have the proper mechanisms in place to support seniors' ability to access a nutritious diet.

At the exosystem level, the participants identified several policies and programs such as OAS, CPP, and Pharmacare that influenced access to food. Consistent with findings of our previous research [1], participants described the protective effect of the GIS, the OAS program for low-income seniors. If a low-income senior has no other source of income besides the OAS basic pension (i.e., did not pay into CPP or have a private pension plan), they can receive up to an additional $738.96 (effective July 2012) in monthly income on top of the basic OAS pension [34], this additional GIS benefit is nontaxable income. As of July 2012, the average monthly GIS benefit received by a single senior is $492.23 [35]. Canada's universal health care has a protective effect on the food security status of seniors. In NS, no senior need absorb the full cost of their medications because of the compulsory drug insurance plan, Pharmacare. Recipients of the GIS also do not have to pay the annual premium associated with the insurance plan. Although Pharmacare is an exosystem factor its existence speaks to Canadians' values and beliefs at the macrosystem level that healthcare should be accessible to every citizen [36]. 

Unfortunately, a recent evaluation of the GIS shows that many seniors who are eligible to receive GIS are not doing so; it is speculated that a large proportion of eligible nonrecipients consists of seniors in vulnerable communities, such as aboriginal people, homeless or near homeless, and immigrants [23]. While increasing the accessibility of GIS would help to address food insecurity among vulnerable seniors, the findings from our provincial food costing research consistently show that even with the GIS, not a great deal of money is left at the end of the month, which can leave lone seniors vulnerable to food insecurity if emergency or unforeseen expenses arise [1, 37, 38]. 

Also at the exosystem level, is the protective role subsidized housing plays on freeing up money to be made available for food. Seven of the eight participants lived in subsidized housing; affordable and accessible housing is necessary to help low-income persons, including seniors, achieve food security [39]. Our findings suggest that housing costs absorb the “lion's share” of seniors' monthly pensions; this has been shown in other research in NS [1, 37] and elsewhere [38]. Finding and maintaining housing on limited income can be a major challenge for seniors, as heard in two of the stories from the women interviewed who spoke of excessively long wait lists to get an apartment in a seniors' manor in urban NS. In 2006, 4% of seniors in NS were able to access the provincial government's Seniors Rental Housing program [4]; many more seniors would benefit from the income-protection subsidized housing offers but there are not enough spaces allocated for the 11% of seniors (13,715), living below the low-income cutoff [4]. It could be speculated that the voices of seniors paying market rent would reflect more food insecure experiences than the women interviewed here.

At a more local level, social supports for seniors may be dwindling. Our qualitative results suggest that reliable and accessible transportation is an issue for senior women in urban NS. Statistics Canada shows that relatively few seniors use public transit [40], and our participants clearly shared their challenges with transit systems. Most seniors in NS live in areas without public transit [4] meaning volunteer drivers, private transportation services, and reliance of family and friends become more and more important to help gain access to groceries. Unfortunately, the trend in NS is migratory as youth and adults leave the province in search of employment [41] and as the volunteer sector dwindles [42]. It is quite plausible the NS senior's microsystem is shrinking as the structures with which the senior has direct contact become fewer and fewer [43]. This is of concern in light of the “world view,” we notice today's seniors working within; will seniors in need reach out to community resources if they've been raised to weather hard times? Who, if not a family member, will notice a lone senior women stretching or skipping meals, or eating nutritionally inadequately? 

The finding that seniors may be experiencing food insecurity, relying heavily on social networks and personal coping strategies suggests existing data of food insecurity in seniors may not be comprehensive; current indicators and measures do not capture the whole experience of how seniors experience food insecurity. In addition, with their generational lens and experience of previous hardships such as the Great Depression and World Wars, food insecurity amongst seniors is not viewed as a serious problem by seniors themselves. This may help to explain why rates of food insecurity among seniors in Canada are reported to be low (3.2%) [44], while poverty rates as determined by the LICO are much higher (11.8%) [45]. It is unclear whether food insecurity among seniors is truly being captured by current means of data collection. If the results from this study are any indication,there may be a great many seniors with similar generational views who are silently “coping”, effectively rendering the issue of food insecurity among seniors invisible. 

 The results from this study are timely, as in Canada, policies in place to protect vulnerable populations such as seniors may be at risk. During the announcement of Canada's Economic Action Plan 2012 (the Federal Budget), significant changes to the OAS program were proposed: starting in 2023, the age of entitlement for all OAS benefits would gradually increase by two years from 65 to 67, with full implementation by 2029. This proposed policy change may mean more seniors will be relying on provincial social assistance and disability programs longer, especially given that statistics are showing that the Canadian population is aging, with population projections suggesting that by 2051 seniors will form 24.7% of the Canadian population compared to 14.4% in 2011, and 9.6% in 1981 [46]. In light of this, our findings raise concerns that the risk of poverty and food insecurity among lone senior women may be significant into the future. 

These findings and our earlier work [1] suggest that OAS and GIS benefits are essential to ensuring food security for seniors. However, even with these public pensions, seniors incorporate a variety of strategies to ensure access to food, as observed by the eight women interviewed. Given the anticipated increase in the aging population in Canada, as well as the changing *world view* of the next generation, the coping strategies employed by these women may be lost and thus, food insecurity rates in future generations will likely increase. This demonstrates the importance of ensuring that progressive and sustainable social policies are implemented at multiple levels. 

We close with the following recommendations to better monitor and address food insecurity in the Canadian senior population; however, lessons learned can be extended beyond our political borders.

- Currently, tools used to capture the prevalence of food insecurity, for example, the Food Security Survey Module used as part of the Canadian Community Health Survey since 2004 only use *income* security as an indicator for *food* security. Other indicators such as health status, social inclusion, availability of affordable and accessible housing and transportation are important influencers on seniors' ability to achieve food security and therefore measurement tools which incorporate consideration of these factors should be developed and validated.
- Adequate funding should be allocated to community programs that provide nutritious food to seniors (e.g., meals on wheels, etc.) to better protect those seniors from malnutrition who are unable to cook or travel due to health/mobility limitations and those with unavailable personal social supports.
- Accessible and affordable housing (e.g., subsidized housing) must be available and reflective of the level of need among community dwelling seniors.
- Accessible municipal transportation, with adequate scheduling and routes, should be available in urban centres, with various methods of community input available to ensure the system is meeting the needs of its users/community residents.
- In terms of further reaching policy implications, federal and provincial governments in Canada should develop and implement a poverty reduction strategy that aims to lift people out of poverty. In relation to seniors, this would include continuing to review and implement changes to public pension systems to ensure income adequacy among Canadian seniors and increasing access and awareness of programs, services, and support, such as Guaranteed Income Supplement for low-income seniors.

## Endnotes

1. The Old Age Security (OAS) program is the cornerstone of Canada's retirement income system. At age 65, it provides seniors with a modest pension (average of $510.17/month in July 2012) [35] if they have lived in Canada for at least 10 years [47].
2. Nearly all Canadians between the ages of 18–70 years who are employed (including self-employed individuals) earning more than $3500 a year contribute to the CPP and are entitled to a retirement pension once they turn 60 years of age [48]. The age at which a senior decides to start collecting his/her pension affects the amount payable to the contributor.
3. Food costing, conducted in towns and cities across Canada, involves using the National Nutritious Food Basket to monitor the cost and affordability of a basic nutritious diet [49].
4. The LICO is an income threshold below which a family will devote 20% more of its income on the necessities of food, shelter, and clothing than the average Canadian family living in a similar-sized community [50].
5. The Guaranteed Income Supplement (GIS) is a part of the Old Age Security (OAS) benefits for lower-income seniors. To be eligible, seniors must be receiving an OAS basic pension and meet other certain income requirements. The monthly amount available depends on the applicant's marital status and income. Seniors must apply for their GIS, generally through the income tax system, and this benefit is not taxable [51].
6. The Nova Scotia Seniors Pharmacare Program is a provincial drug insurance program that helps seniors with the cost of their prescription drugs. GIS recipients are not required to pay the annual premium [52].
